# Overexpressed transferrin receptor implied poor prognosis and relapse in gastrointestinal stromal tumors

**DOI:** 10.3389/fonc.2023.1151687

**Published:** 2023-08-22

**Authors:** Chun Zhuang, Xiaoqi Li, Linxi Yang, Xinli Ma, Yanying Shen, Chen Huang, Tao Pan, Jianzhi Cui, Bo Ni, Ming Wang

**Affiliations:** Department of Gastrointestinal Surgery, Renji Hospital, Shanghai Jiao Tong University School of Medicine, Shanghai, China

**Keywords:** gastrointestinal stromal tumor (GIST), ferroptosis, TFRC, tumor biomarkers, prognosis

## Abstract

Ferroptosis, as a novel-induced programmed cell death, plays critical roles in the pathogenesis of cancers. However, the promising biomarkers of ferroptosis in gastrointestinal stromal tumor (GIST) remain to be elucidated. Herein, the expression of ferroptosis-related genes was analyzed in GIST. Among the 64 ferroptosis-related genes, transferrin receptor (TFRC) expression presented a remarkable upregulation in high-risk patients through Gene Expression Omnibus (GEO) dataset analysis, as well as its significant change after imatinib was treated. Kyoto Encyclopedia of Genes and Genomes (KEGG) pathway enrichment analysis of TFRC-relevant genes revealed that TFRC expression was closely associated with cell growth pathways and metabolism-related pathways. Furthermore, patients at high risk of recurrence were more likely to exhibit high TFRC expression by immunohistochemistry. Additionally, high TFRC expression indicated an undesirable state of patient relapse, which could serve as a powerful significant independent predictor of recurrence-free survival (RFS). In summary, we systematically summarize the expression characteristics and clinical relevance of TFRC and show that TFRC can be used as a prognostic factor, which can be considered a potential therapeutic target in GIST.

## Introduction

Gastrointestinal stromal tumor (GIST) is a class of mesenchymal neoplasms of the digestive tract, and most of them possess an activated mutation of KIT or platelet-derived growth factor receptor alpha (PDGFRA) (1). People over 50 years have a high incidence of suffering from GIST, and an increasing incidence of GIST occurs among younger people (2). Until now, surgical resection remains the primary therapeutic regimen for GIST. Although imatinib mesylate was approved for first-line treatment and yields significant improvement in survival for unresectable and metastasized GIST patients since 2002, the majority of them would suffer disease progression after treatment for 2–3 years (2). There are no alternative treatments available to treat them. Hence, it is urgently required to develop novel biomarkers and molecule targets against GIST.

Ferroptosis, as a novel-induced programmed cell death, is characterized by the accumulation of intracellular iron and lipid reactive oxygen species (ROS) (3). Recently, extensive studies were reported on cancer initiation, progression, or drug sensitivity, which showed great promise in cancer treatment (4, 5). For example, erastin, a ferroptosis inducer, showed a significant synergistic effect on the effect of antitumor therapy when combined with cisplatin (6).

In addition, emerging evidence suggested that aberrant expression of ferroptosis-related genes was closely related to clinical characteristics. SLC7A11, a core target-regulating ferroptosis, is frequently overexpressed in most tumors, such as colon adenocarcinoma (COAD), lung adenocarcinoma (LUAD), and esophageal cancer (ESCA), which would affect lymphatic metastasis, the infiltration of immune cells, etc. (7). Additionally, SLC3A2, VDAC2, and SLC7A11 expression increased continuously with the TNM stage, and FTH1 and LPLCAT3 expression increased continuously with pathological grade in pan-cancer (8). However, the expression pattern of ferroptosis-related genes and the association between them and clinical relevance remain largely unknown in GIST.

In this study, data mining of ferroptosis-related genes using the Gene Expression Omnibus (GEO) dataset confirmed that transferrin receptor (TFRC) expression was aberrant in high-risk patients and would be affected by imatinib mesylate treatment. We then investigated the enriched signaling pathways by TFRC driving. Next, we validated its expression and evaluated its clinical relevance, as well as the prognostic value in the collected GIST patient cohorts.

## Materials and methods

### Data mining

All original data were downloaded from GEO (https://www.ncbi.nlm.nih.gov/geo/) databases. Gene set enrichment analysis (GSEA) was performed to investigate the differences in signaling pathways involved by TFRC, with the gene set “c2.cp.kegg.v6.2.symbols.gmt” as the reference.

### Patient characteristics and ethics

In total, 587 samples containing detailed clinical prognostic information of pathologic diagnosis of GIST were obtained from pathology files, which were treated in the Department of General Surgery, Ren Ji Hospital, School of Medicine, Shanghai Jiao Tong University. Informed consent forms were signed by all patients. The clinical information including age at diagnosis, gender, modified National Institutes of Health (NIH) criteria, tumor size, mitotic figures, recurrence, and overall survival (OS) state is summarized in **Table 1**. Preoperative imaging data and surgery records were confirmed to ensure that the included patients had resected localized GISTs. Patients with unresectable or metastatic GISTs or other malignant tumors were excluded. Tissue microarray (TMA) was made using the 587 formalin-fixed paraffin-embedded tumor biopsies. All samples were collected under institutional review board approval. The study was approved by the Research Ethics Committee of Ren Ji Hospital and carried out in accordance with ethical standards as formulated in the Declaration of Helsinki, with ethical approval number 2018-029.

**Table 1:** Patients’ characteristics.

Clinicopathological factors		n (%)
**Age**	≤61	298 (50.77)
	>61	289 (49.23)
**Gender**	Male	294 (50.09)
	Female	293 (49.91)
**Modified NIH criteria**	Low risk	254 (43.27)
	Intermediate risk	95 (16.18)
	High risk	238 (40.55)
**Tumor size**	≤2 cm	51 (8.69)
	2–5 cm	247 (42.08)
	5–10 cm	212 (36.11)
	>10 cm	77 (13.12)
**Mitotic figures**	≤5	442 (75.30)
	5–10	75 (12.78)
	>10	70 (11.92)
Recurrence	No	513 (87.39)
	Yes	74 (12.61)
**Overall survival state**	Alive	556 (94.72)
	Dead	31 (5.28)

NIH, National Institutes of Health.

### Immunohistochemistry

**Figure 1:**
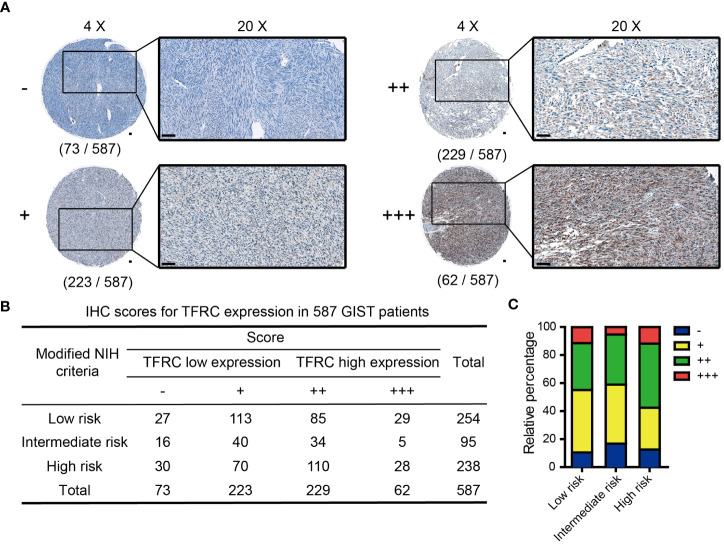
IHC scores of TFRC expression in 587 GIST patients. **(A)** Representative IHC score for TFRC expression in GIST tissues (original magnification: ×4 and ×20, round panel and orthogonal panel, respectively; scale bars, 100 μm). **(B)** IHC score distribution of TFRC in 587 cases. **(C)** Statistical analysis of the IHC score distribution in patients with different degrees of risk. IHC, immunohistochemistry; GIST, gastrointestinal stromal tumor.

The protocol for immunohistochemistry (IHC) was performed according to a previous description (9). The prepared TMA sections were dewaxed with xylene and hydrated with alcohol. Sodium citrate was used for antigen retrieval, and hydrogen peroxide was used to block endogenous peroxidase. Bovine serum albumin (BSA) was used to block non-specific sites. All sections were incubated with an appropriate primary antibody and secondary antibody. The primary antibody used was TFRC (Proteintech, Rosemont, IL, USA; 10084-2-AP, 1:400). TFRC expression was classified semi-quantitatively, which was independently scored by two pathologists blinded to clinical outcomes, and differences were resolved by mutual agreement, as previously described (10, 11). Briefly, the score of TFRC expression was assigned semi-quantitative terms, namely, “−”, “+”, “++”, or “+++”: “−” = “none” (no staining), “+” = “weak staining”, “++” = “moderate staining”, or “+++” = “strong staining” (**Figure 1A**). “++” and “+++” were considered as higher expression, and the others were considered as lower expression. 

### Statistical analyses

Data are shown as means ± SD. GraphPad Prism 5 software was used to calculate cumulative survival time by the Kaplan–Meier method *via* the log-rank test or Cox regression analysis, as shown by the Kaplan–Meier (KM) curve. Fisher’s exact test and chi-square test were used for comparison between groups through SPSS 20.0 (Chicago, IL, USA). Correlation between markers was obtained using Spearman’s correlation method. All tests were two-sided except as indicated, and p < 0.05 was considered statistically significant.

## Results

### Identification of TFRC as a key upregulated ferroptosis gene in GISTs

**Figure 2:**
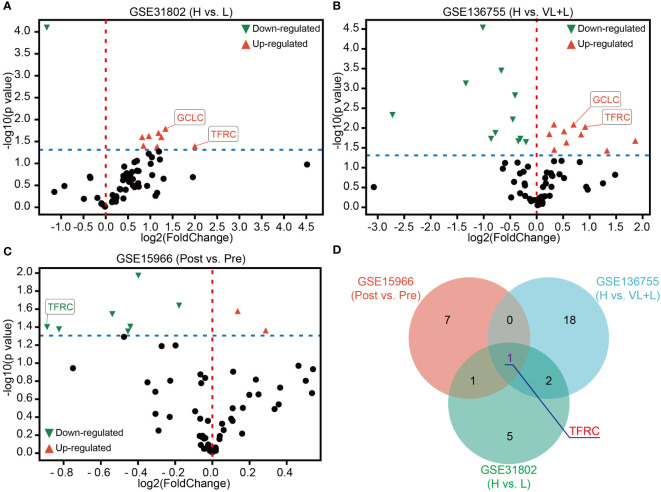
Identification of TFRC as a key ferroptosis association in GIST. **(A, B)** Volcano plots of differentially expressed ferroptosis-associated genes in GSE31802 and GSE136755 between high- and low- or very low-risk patients. **(C)** Volcano plots of differential expression of ferroptosis-associated genes before and after treatment with IM in GSE15966. **(D)** Venn diagram showing that TFRC was the common dysregulated gene of ferroptosis association in GSE31802, GSE136755, and GSE15966. p-Value < 0.05. Green, downregulated genes; red, upregulated genes. IM, imatinib mesylate.

Ferroptosis, as a novel-induced programmed cell death, has been widely researched in cancers. Hence, 64 genes assigned to ferroptosis pathways in the MSigDB database were gathered (**Supplementary Table 1**). To determine the potential dysregulated ferroptosis association in GIST, two independent GEO datasets (GSE31802 and GSE136755) containing high- and low-risk patients were utilized. As shown in **Figures 2A, B**, many ferroptosis-associated genes were significantly up- or downregulated in high-risk compared to low-risk patients. In addition, only GCLC and TFRC with obvious upregulation were obtained by the overlapping analysis of these two gene sets (**Supplementary Figures 1A, B**). A gradient increased expression pattern of GCLC and TFRC was demonstrated with the increase in risk level (**Supplementary Figures 1C, D**). As known to us, imatinib mesylate (IM) has been used as a major adjuvant treatment for advanced GIST patients (12). Here, we were more interested in determining potentially dysregulated ferroptosis association in GISTs with IM treatment. As shown in **Figure 2C**, multiple genes displayed significant up- or downregulation in patients after IM treatment compared to that before IM treatment analyzed in GSE15966. Finally, TFRC was selected for further research *via* overlapping analysis of these three gene sets (**Figure 2D**).

### Exploration of the potential molecular pathways associated with TFRC in GIST

**Figure 3:**
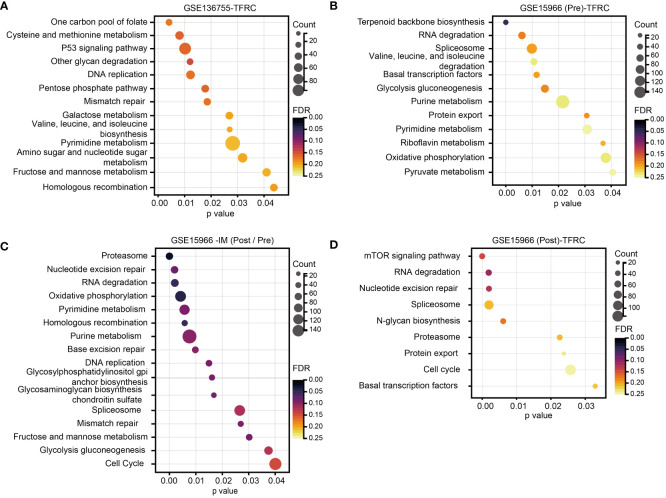
Analysis of TFRC-involved signaling pathways in GIST. **(A, B)** GSEA for the signaling pathways involved in the high TFRC expression subpopulation of GSE136755, as well as the high TFRC expression subpopulation GSE15966 pre-IM treatment. **(C)** GSEA for the signaling pathways involved in IM treatment in GSE15966. **(D)** GSEA for the signaling pathways of TFRC participants after IM treatment in GSE15966. p-Value < 0.05, FDR < 0.25. GIST, gastrointestinal stromal tumor; GSEA, gene set enrichment analysis; IM, imatinib mesylate; FDR, false discovery rate.

To further study the signaling pathways involved in TFRC in GIST, we conducted GSEA in the GEO dataset. Through the cutoff values of p-value < 0.05 and false discovery rate (FDR) < 0.25, we determined that high TFRC expression was positively correlated to the P53 signaling pathway, DNA replication, Pentose phosphate pathway, Mismatch repair, Pyrimidine metabolism, etc., in GSE136755 (**Figure 3A**), and Glycolysis gluconeogenesis, Purine metabolism, Pyrimidine metabolism, Oxidative phosphorylation, Pyruvate metabolism, etc., in GSE15966 pre-IM treatment (**Figure 3B**). These data displayed that TFRC was mainly involved in metabolism-related signaling pathways to exert an oncogenic role in GIST. Additionally, we further analyzed the potential signaling pathways involved in TFRC with IM treatment in GIST. First, we found there were remarkable interactions between IM treatment with cell growth pathways (such as Nucleotide excision repair, Base excision repair, DNA replication, Mismatch repair, and Cell Cycle) and metabolism-related pathways (Oxidative phosphorylation, Pyrimidine metabolism, Purine metabolism, and Glycolysis gluconeogenesis) (**Figure 3C**). Meanwhile, there were remarkable interactions of TFRC with the mTOR signaling pathway, Nucleotide excision repair, and cell cycle in GSE15966 post-IM treatment (**Figure 3D**). Overall, TFRC plays an important role in GIST progression, as well as in the process of IM treatment.

### Analysis of TFRC expression and its association with clinical characteristics in GIST

To evaluate TFRC expression levels, IHC was performed in a set of TMAs containing 587 GIST samples. According to the staining score of TFRC, GIST patients were clustered into high and low TFRC expression subpopulations. As shown in **Figure 2A**, 291 cases were divided into a high group (scores of “++” and “+++”) and others into a low group (scores of “−” and “+”; **Figure 1A**). Consistent with the analyzed results by the GEO dataset, remarkably more patients possessed higher TFRC expression in high-risk subpopulations (**Figures 1B, C**).

### Prognostic significance of TFRC in GIST

**Figure 4:**
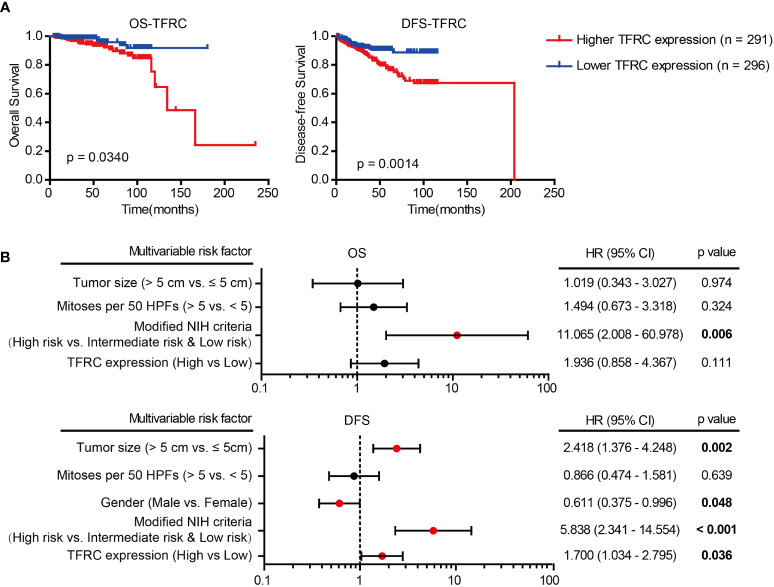
Prognostic significance of TFRC. **(A)** Kaplan–Meier analyses of overall survival (OS; p = 0.0340) and relapse-free survival (RFS; p = 0.0014) of GIST patients in correlation with high or low TFRC expression. **(B)** Multivariate Cox regression analysis of OS and RFS performed in GIST patients. GIST, gastrointestinal stromal tumor; OS, overall survival; RFS, recurrence-free survival.

Subsequently, the prognostic value of TFRC in GISTs was confirmed. As depicted in **Table 2**, clinical association analyses revealed that in addition to a positive correlation with modified NIH criteria, upregulation of TFRC expression was also associated with most relapsed patients. Moreover, Kaplan–Meier analyses showed that TFRC negatively correlates with OS and recurrence-free survival (RFS) in GIST patients (**Figure 4A**). Next, Cox proportional hazards models were applied to analyze the relationship between TFRC expression and patient outcomes. Univariate analyses showed that TFRC expression, NIH risk degree, mitotic figures, and tumor size were significantly correlated with OS (**Table 3**) and RFS (**Table 4**). However, multivariate analyses showed that TFRC expression was merely an independent prognostic predictor for RFS (**Figure 4B**). These findings indicate that TFRC might be a key ferroptosis gene, which is commonly upregulated and associated with relapse in high-risk GISTs.

**Table 2:** Correlations between TFRC expression and clinicopathological features in GISTs.

Clinicopathological feature		Expression of TFRC		p-Value (χ^2^ test)
		Low (n = 296, 50.43%)	High (n = 291, 49.57%)	
**Age**	≤61	139	159	0.063
	>61	157	132	
**Gender**	Male	146	148	0.710
	Female	150	143	
**Modified NIH criteria**	Low risk	140	114	**0.003**
	Intermediate risk	56	39	
	High risk	100	138	
**Tumor size**	≤2 cm	28	23	0.311
	2–5 cm	131	116	
	5–10 cm	105	107	
	>10 cm	32	45	
**Mitotic figures**	≤5	234	208	0.085
	5–10	34	41	
	>10	28	42	
Recurrence	Yes	23	51	**<0.001**
	No	273	240	

GIST, gastrointestinal stromal tumor; NIH, National Institutes of Health. P-values < 0.05 are indicated using bold font

**Table 3:** Univariate analyses of prognostic parameters for OS in GISTs.

Prognostic parameter	Univariate analysis		
	HR	95% CI	p-Value
**Expression of TFRC (high *vs.* low)**	2.352	1.042–5.307	**0.039**
**Age (>61 *vs.* ≤ 61)**	0.514	0.242–1.090	0.083
**Gender (male *vs.* female)**	0.753	0.368–1.540	0.437
**Modified NIH criteria (high risk *vs.* intermediate risk and low risk)**	15.314	3.626–64.680	**<0.001**
**Tumor size (>5 cm *vs.* ≤ 5 cm)**	3.826	1.448–10.111	**0.007**
**Mitotic figures (>5 *vs.* ≤ 5)**	4.034	1.899–8.567	**<0.001**
**Recurrence (yes *vs.* no)**	2,305.866	0.787–6,751,922.497	0.057

OS, overall survival; GIST, gastrointestinal stromal tumor; NIH, National Institutes of Health. P-values < 0.05 are indicated using bold font

**Table 4:** Univariate analyses of prognostic parameters for DFS in GISTs.

Prognostic parameter	Univariate analysis		
	HR	95% CI	p-Value
**Expression of TFRC (high *vs.* low)**	2.192	1.337–3.593	**0.002**
**Age (>61 *vs.* ≤ 61)**	0.731	0.461–1.160	0.184
**Gender (male *vs.* female)**	0.511	0.315–0.829	**0.007**
**Modified NIH criteria (high risk *vs.* intermediate risk and low risk)**	10.208	5.080–20.512	**<0.001**
**Tumor size (>5 cm *vs.* ≤ 5 cm)**	2.785	1.650–4.701	**<0.001**
**Mitotic figures (>5 *vs.* ≤ 5)**	6.061	3.737–9.832	**<0.001**

OS, overall survival; GIST, gastrointestinal stromal tumor; NIH, National Institutes of Health. P-values < 0.05 are indicated using bold font

P-values < 0.05 are indicated using bold font.

### The sensitivity and specificity of TFRC for RFS in GISTs

**Figure 5:**
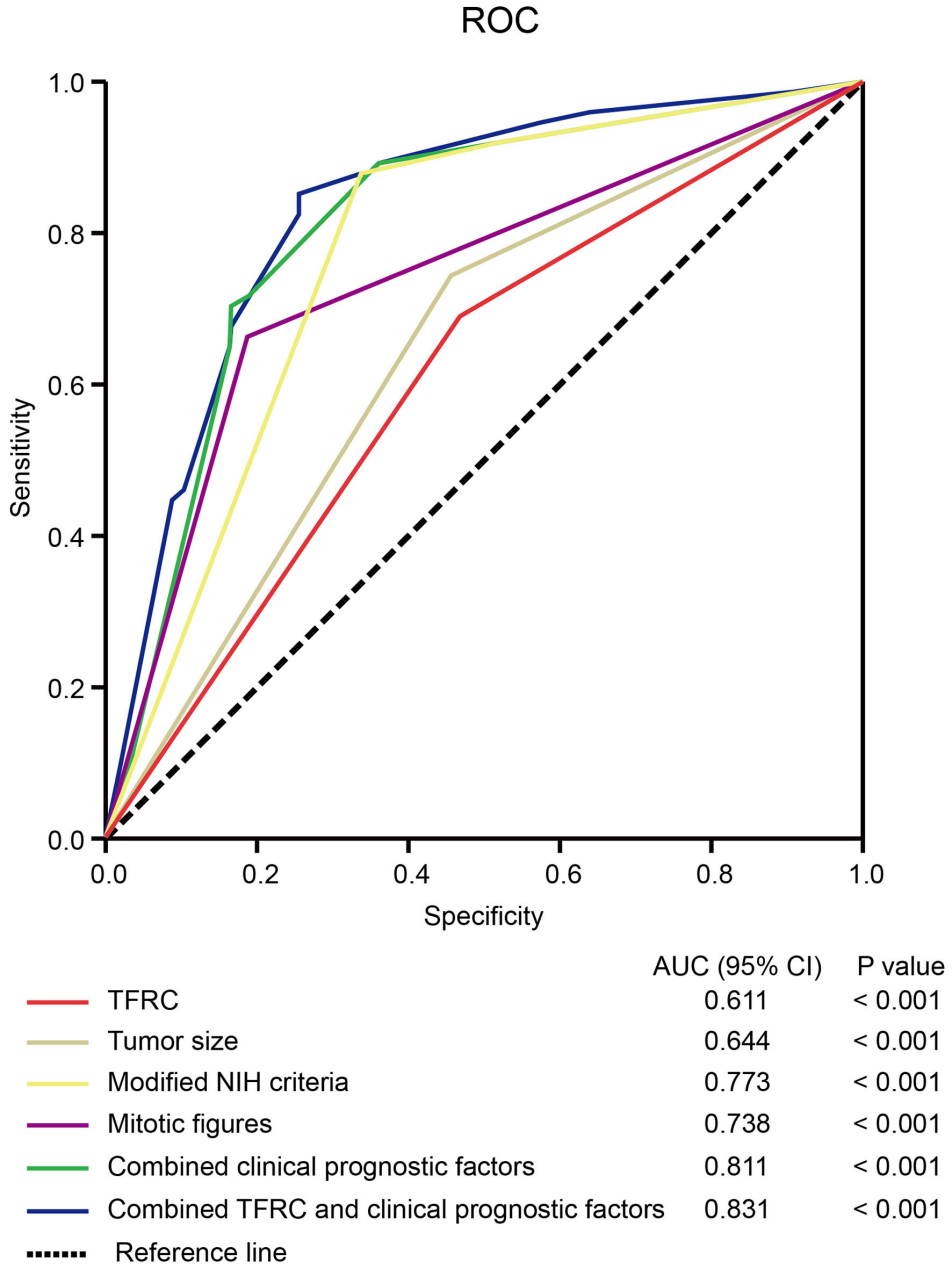
The sensitivity and specificity of TFRC for DFS. The ROC curve compares the prognostic accuracy of TFRC with clinicopathological features in all 587 GIST patients by logistic regression. ROC, receiver operating characteristic; AUC, area under the curve. Combined clinical prognostic factors include modified NIH criteria, tumor size, and mitotic figures. DFS, disease-free survival; GIST, gastrointestinal stromal tumor; NIH, National Institutes of Health. Note. DFS, disease-free survival; GIST, gastrointestinal stromal tumor; NIH, National Institutes of Health.

To further confirm the prognostic accuracy of TFRC in GIST, logistic regression was conducted to compare the sensitivity and specificity of TFRC for RFS. Multiple models were constructed, including TFRC as a single clinicopathological feature, combinations of clinicopathological features, and TFRC combined with clinicopathological features **Figure 5**. A receiver operating characteristic (ROC) curve comparison demonstrated that the area under the curve (AUC) for TFRC combined with other clinicopathological features was higher than that for any other single or combined factor (AUC = 0.831, p < 0.001). These results indicated that TFRC combined with other clinicopathological features had greater sensitivity and specificity and was a stronger RFS predictor than any single risk factor or their combination.

## Discussion

Ferroptosis, a newly defined form of programmed cell death, is characterized by iron overload, lipid ROS accumulation, and lipid peroxidation. Extensive studies in cancers have shown the intimate association between ferroptosis with cancer initiation and progression. For example, SLC7A11, a core target-regulating ferroptosis, is overexpressed and is correlated with worse survival in non-small cell lung cancer (NSCLC) (13) and pancreatic ductal adenocarcinoma (PDAC) (14) patients, which is classified as a suppressor of ferroptosis. However, few studies about the primary mechanisms and signal pathways relevant to ferroptosis have been performed, as well as their potential roles in GIST. Therefore, our study aimed to reveal the clinical significance of ferroptosis-related genes in GIST.

First, data mining using GEO datasets displayed that overexpressed TFRC exists in high-risk patients. However, the clinical relevance of TFRC expression and its prognostic value for GIST patients remain unclear. Here, we found that increased TFRC expression was closely related to tumor relapse and poor prognosis, which suggested that TFRC has a role in carcinogenesis. However, its detailed function and underlying mechanism in GIST remain unclear and warrant further studies.

TFRC, also known as CD71, is one of the most important receptor-mediated controls during the iron intake process in generic cells *via* binding with iron–transferrin complex to facilitate iron into cells. Meanwhile, TFRC has been verified to be abnormally expressed in various cancers (15). Dramatically increased TFRC expression was associated with a worse prognosis in epithelial ovarian cancer, which would accelerate the progression *via* upregulation of AXIN2 expression (16). CD71-positive cells enriched by HPV-E6 protein promoted cancer aggressiveness in cervical cancer cells (17). In hepatocellular carcinoma (HCC), O-GlcNAcylation can increase ferroptosis sensitivity *via* transcriptional elevation of TFRC to increase the iron concentration in cells (18). In general, inducing ferroptosis in tumor cells can effectively inhibit tumor growth (19). Moreover, TFRC was categorized into ferroptosis drivers by summarizing ferroptosis regulators by Kyoto Encyclopedia of Genes and Genomes (KEGG) pathway enrichment and selecting genes using the FerrDb database, PubMed, and Google Scholar (8). Hence, more in-depth studies need to be conducted to verify the role of TFRC on ferroptosis in GIST. Although TFRC is an important iron uptake receptor in cancer cells, its functions and mechanisms in ferroptosis and tumor progression remain unclear. Therefore, TFRC may not mediate GIST progression by regulating ferroptosis, and other pathways may be involved, such as the metabolism-related pathway shown in **Figure 3**, which suggests that TFRC is tissue and tumor type. Several studies in gastric cancer (GC) have shown that the expression of TFRC is reversely correlated with a poor prognosis in primary GC (20). Xiaojing Cheng et al. (21) conducted experiments both *in vitro* and *in vivo*, revealing that TFRC-negative cells exhibit properties of tumor-initiating cells and possess immune escape features. Further investigation into the effects of TFRC on GIST cells could offer potential therapeutic strategies for patients with GIST.

Moreover, data mining also revealed that decreased expression of TFRC occurred after treatment with imatinib, which indicates its relationship with drug sensitivity. In addition, clinical sample analysis revealed a positive correlation between TFRC expression and patient relapse. Survival analysis was indicative that patients with high TFRC expression possessed more undesirable disease-free survival (DFS) outcomes than those with low TFRC expression. These data are consistent with the analyzed results in previous reports of other cancers, which further supported the pivotal role in cancer progression. Previous research has revealed that the CD71^+^ subpopulation of cervical cancer cells exhibited enhanced resistance to irradiation and suppression of CD71-inhibited sensitized cells to irradiation treatment (17). Furthermore, more frequently upregulated TFRC expressions occur in some drug-resistant human cancer cells (22), therefore requiring more in-depth studies to clarify the role of TFRC in the sensitivity and resistance of cancer cells to imatinib in GIST therapy. Targeting TFRC using compounds such as curcumin (one of the most successful chemo-preventive compounds) to intervene with the progression of cancers seems feasible (23). Moreover, increasing the concentrations of TFRC-targeted superparamagnetic iron oxides in tumor tissues *via* magnetic fields could inhibit tumor progression, which is a promising cancer treatment (24, 25). However, whether TFRC can be an effective target for GIST treatment needs more theoretical support.

In summary, our study found that the expression of TFRC was significantly upregulated in high-risk GIST and resulted in a higher relapse rate. Meanwhile, decreased expression of TFRC caused by imatinib treatment occurred. Therefore, developing new treatments targeting TFRC would be a potential therapeutic approach in GIST, as well as its combination with imatinib.

## Data availability statement

The original contributions presented in the study are included in the article/**Supplementary Material**. Further inquiries can be directed to the corresponding authors.

## Ethics statement

The studies involving humans were approved by Shanghai Jiaotong University School of Medicine, Renji Hospital Ethics Committee. The studies were conducted in accordance with the local legislation and institutional requirements. The participants provided their written informed consent to participate in this study.

## Author contributions

MW provided the whole study design. BN supervised the experiment conduction. CZ and XL carried out the experiments and organized the manuscript. LY finished the bioinformatics analysis. XM and YS collaborated to collect the specimens and record clinical information. CH, TP, and JC were responsible for statistical analysis. MW and BN offered critical reviews. All authors contributed to the article and approved the submitted version.

## Supplementary material

Supplementary Figure 1 Aberrantly expression of ferroptosis-associated genes in GIST. (A) Venn diagram showed the overlap of significantly down-regulated ferroptosis-associated genes in GSE31802 and GSE136755. (B) Venn diagram showed the overlap of significantly up-regulated ferroptosis-associated genes in GSE31802 and GSE136755. (C) Expression analysis of GCLC in different risk degree patients (p = 0.0041). (D) Expression analysis of TFRC in different risk degree patients (p = 0.0041). H: High risk, Inter: Intermediate risk, L: Low risk, VL: Very low risk.
