# Supplementary figures and images for: Overexpressed transferrin receptor implied poor prognosis and relapse in gastrointestinal stromal tumors
Source: Front Oncol. 2023 Aug 22;13:1151687. doi: 10.3389/fonc.2023.1151687 (PMC10477977; doi:10.3389/fonc.2023.1151687)

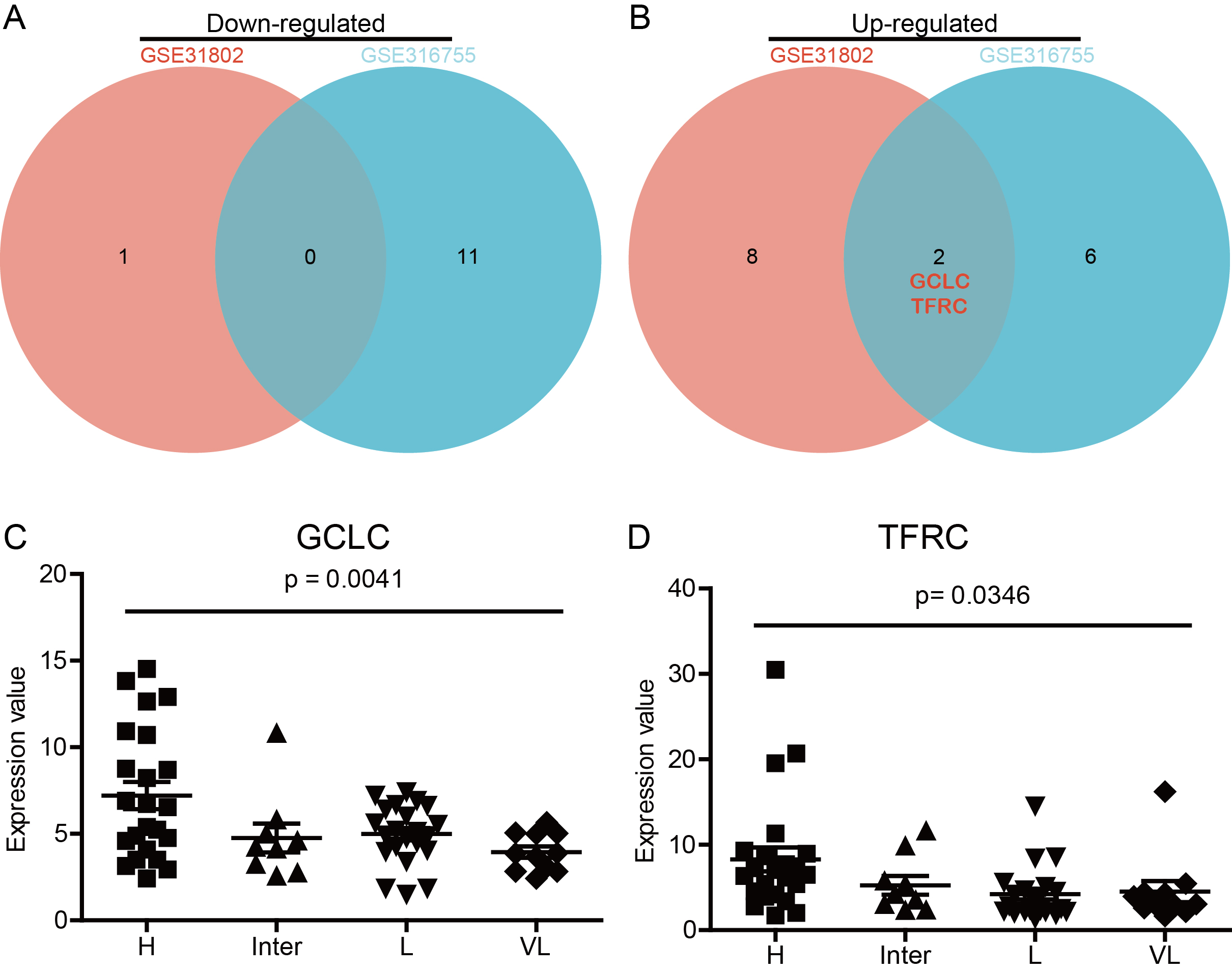

Supplement: Supplementary Figure 1 — Aberrantly expression of ferroptosis-associated genes in GIST. (A) Venn diagram showed the overlap of significantly down-regulated ferroptosis-associated genes in GSE31802 and GSE136755. (B) Venn diagram showed the overlap of significantly up-regulated ferroptosis-associated genes in GSE31802 and GSE136755. (C) Expression analysis of GCLC in different risk degree patients (p = 0.0041). (D) Expression analysis of TFRC in different risk degree patients (p = 0.0041). H: High risk, Inter: Intermediate risk, L: Low risk, VL: Very low risk. [file Image_1.jpeg]
