# Supplementary material for: Overexpressed transferrin receptor implied poor prognosis and relapse in gastrointestinal stromal tumors
Source: Front Oncol. 2023 Aug 22;13:1151687. doi: 10.3389/fonc.2023.1151687 (PMC10477977; doi:10.3389/fonc.2023.1151687)
Supplement: Supplementary file 2 [file Table_1.docx]

**Supplementary table.1 Relative gene of Ferroptosis**

| ACSL1 | AKR1C3 | CISD1 | FTL | HMOX1 | NOX1 | SAT2 | SLC7A11 |
| --- | --- | --- | --- | --- | --- | --- | --- |
| ACSL3 | ALOX15 | COQ2 | FTMT | HSPB1 | NOX4 | SLC11A2 | STEAP3 |
| ACSL4 | ATG5 | CP | GCH1 | IREB2 | PCBP1 | SLC1A5 | TF |
| ACSL5 | ATG7 | CTH | GCLC | LPCAT3 | PCBP2 | SLC38A1 | TFRC |
| ACSL6 | BACH1 | CYBB | GCLM | MAP1LC3A | PHKG2 | SLC39A14 | TP53 |
| AIFM2 | CBS | DPP4 | GPX4 | MAP1LC3B | POR | SLC39A8 | TXNRD1 |
| AKR1C1 | CHMP5 | FDFT1 | GSS | MAP1LC3C | PRNP | SLC3A2 | VDAC2 |
| AKR1C2 | CHMP6 | FTH1 | HMGCR | NCOA4 | SAT1 | SLC40A1 | VDAC3 |
